# Vascular growth responses to chronic arterial occlusion are unaffected by myeloid specific focal adhesion kinase (FAK) deletion

**DOI:** 10.1038/srep27029

**Published:** 2016-05-31

**Authors:** Joshua L. Heuslein, Kelsey P. Murrell, Ryan J. Leiphart, Ryan A. Llewellyn, Joshua K. Meisner, Richard J. Price

**Affiliations:** 1 Department of Biomedical Engineering, University of Virginia, Charlottesville, Virginia, United States of America; 2 Department of Microbiology, University of Virginia, Charlottesville, Virginia, United States of America; 3 Department of Radiology, University of Virginia, Charlottesville, Virginia, United States of America; 4 Department of Radiation Oncology, University of Virginia, Charlottesville, Virginia, United States of America

## Abstract

Arteriogenesis, or the lumenal expansion of pre-existing arterioles in the presence of an upstream occlusion, is a fundamental vascular growth response. Though alterations in shear stress stimulate arteriogenesis, the migration of monocytes into the perivascular space surrounding collateral arteries and their differentiation into macrophages is critical for this vascular growth response to occur. Focal adhesion kinase’s (FAK) role in regulating cell migration has recently been expanded to primary macrophages. We therefore investigated the effect of the myeloid-specific conditional deletion of FAK on vascular remodeling in the mouse femoral arterial ligation (FAL) model. Using laser Doppler perfusion imaging, whole mount imaging of vascular casted gracilis muscles, and immunostaining for CD31 in gastrocnemius muscles cross-sections, we found that there were no statistical differences in perfusion recovery, arteriogenesis, or angiogenesis 28 days after FAL. We therefore sought to determine FAK expression in different myeloid cell populations. We found that FAK is expressed at equally low levels in Ly6C^hi^ and Ly6C^lo^ blood monocytes, however expression is increased over 2-fold in bone marrow derived macrophages. Ultimately, these results suggest that FAK is not required for monocyte migration to the perivascular space and that vascular remodeling following arterial occlusion occurs independently of myeloid specific FAK.

Peripheral arterial disease (PAD) has become a global problem with an estimated 202 million people with PAD worldwide^1^. PAD occurs when atherosclerotic plaques occlude a major artery, typically in the lower limbs, thereby limiting blood flow to the distal tissue. While surgical and catheter-based revascularization approaches have shown some success, many patients with PAD are poor candidates for these treatment options^2^. Revascularization strategies to stimulate the growth of new capillaries from preexisting vessels (i.e. angiogenesis) or lumenal expansion of pre-existing arteries (i.e. arteriogenesis) remain promising therapeutic options, despite their limited success to date^3^. The stimulation of angiogenesis is important in PAD as capillary density is reduced in these patients^2^,^4^,^5^, however, it is also imperative to restore the driving pressure to the distal tissue via lumenal expansion (i.e. arteriogenesis) of collateral arteries bypassing the occlusion(s)^6^,^7^,^8^.

Arteriogenesis is a multi-faceted, highly coordinated process involving recruitment, migration, and proliferation of multiple cell types as well as the reorganization of the extracellular matrix^3^. In the presence of an arterial occlusion, a significant drop in pressure in the downstream vascular network drives blood flow along a network of pre-existing collateral arteries that bypass the occlusion. The altered hemodynamics through these collateral artery networks leads to increased expression of inflammatory cytokines (e.g. MCP-1)^9^ and cell adhesion molecules (e.g. ICAM-1, VCAM-1)^10^,^11^,^12^,^13^ enabling monocytes to migrate into the perivascular space and transdifferentiate into macrophages^14^,^15^. Perivascular macrophages then secrete cytokines and growth factors that stimulate proliferation of endothelial and smooth muscle cells leading to the outward lumenal growth of collateral arteries^12^,^16^.

In order for this perivascular accumulation to occur, monocytes and macrophages need to migrate through the extracellular matrix. In response to migratory stimuli, they polarize and extend lamellipodia and filopodia in the direction of a chemotactic gradient^17^. Stable attachments near the leading edge of these protrusions form, followed by translocation of the cell body forward, release of cell-matrix adhesions and retraction at the cell rear. The formation and regulation of these structures is controlled by dynamic reorganization of the cytoskeleton and by integrin-mediated interactions with the extracellular matrix, such as focal adhesions^18^,^19^. Major components of focal adhesions are the members of the focal adhesion kinase family, focal adhesion kinase (FAK) and Pyk2. FAK is a cytoplasmic non-receptor tyrosine kinase activated by the autophosphorylation of its Y397 residue following integrin ligation^20^. FAK has been shown to be a key regulator of cell migration as demonstrated by the decreased migratory response of FAK-null fibroblasts in response to chemotactic and hapotactic stimuli^21^,^22^,^23^,^24^. In addition, FAK coordinates lamellipodial formation, turnover, and disassembly of focal adhesions, all required for cell motility^25^,^26^,^27^.

Recently, the critical role of FAK in cell motility has been expanded to primary monocytes and macrophages. Adherent macrophages derived from myeloid-specific FAK knockout mice exhibit impaired focal adhesion turnover, an inability to form stable lamellipodia, and decreased chemotaxis in response to CSF-1, SDF-1α, and MCP-1 *in-vitro*^28^. Moreover, the recruitment of CD11b-positive cells is impaired in conditional FAK knockout mice during a thioglycollate induced inflammatory challenge^28^. Since monocyte and macrophage motility is necessary for arteriogenesis^11^,^29^,^30^,^31^, altering FAK function specifically in myeloid cells, a lineage which contains monocytes and macrophages, could therefore present a powerful tool to understand how myeloid-motility affects arteriogenesis. We hypothesized that a myeloid-specific conditional deletion of FAK would negatively impact vascular remodeling in the mouse femoral arterial ligation model by disrupting monocyte motility.

## Results and Discussion

### Conditional bone marrow macrophage-specific FAK deletion does not affect perfusion recovery after FAL

**Figure 1:**
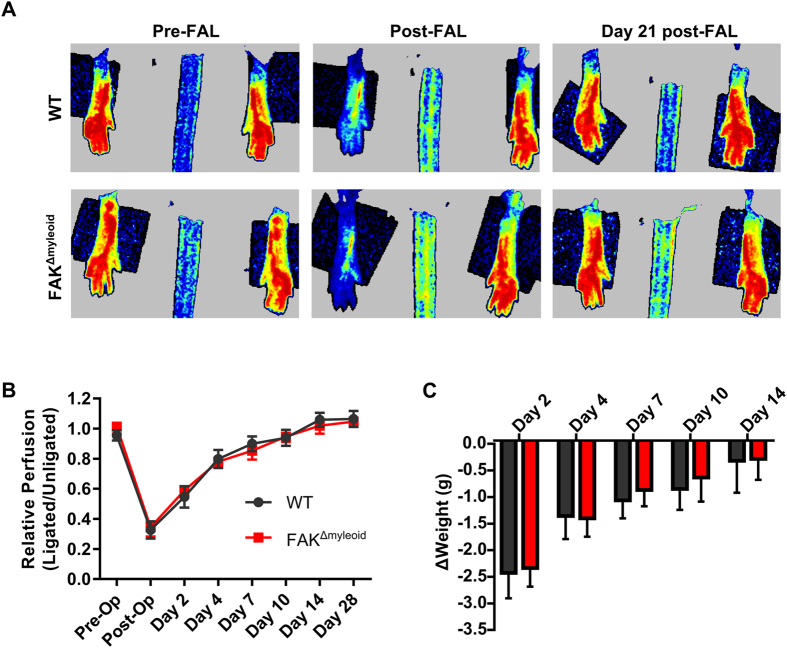
Myeloid-specific conditional FAK deletion does not affect perfusion recovery after FAL. (**A**) Representative images of foot perfusion in wild-type (WT) and FAK^Δmyeloid^ mice as determined by laser Doppler perfusion imaging. (**B**) Laser Doppler perfusion recovery curve (ligated limb normalized to the unligated limb) and (**C**) weight recovery FAK^Δmyeloid^ and age-matched littermate controls (WT) after FAL. There were no statistical differences between FAK^Δmyeloid^ and WT mice (n = 6).

To test this hypothesis, we performed femoral artery ligation to stimulate arteriogenesis on control littermates (WT) and myeloid-specific FAK knockout mice (FAK^Δmyeloid^). Laser Doppler perfusion imaging (LDPI) was used to assess foot perfusion recovery following FAL in FAK^Δmyeloid^ and control mice. Both knockout and control mice show a perfusion deficit only within the first 10 days after FAL with a return to baseline 14 days after FAL (Fig. 1A,B, n = 6 and n = 7, respectively). Postoperative weight loss was used as a measure of gross functional recovery following FAL. Despite the initial drop, weight was fully restored by day 14 in both groups, mirroring the perfusion recovery (Fig. 1C). There were no statistical differences between FAK^Δmyeloid^ or control mice in either foot perfusion or weight recovery.

### Conditional loss of macrophage-specific FAK does not alter arteriogenesis in response to FAL

**Figure 2:**
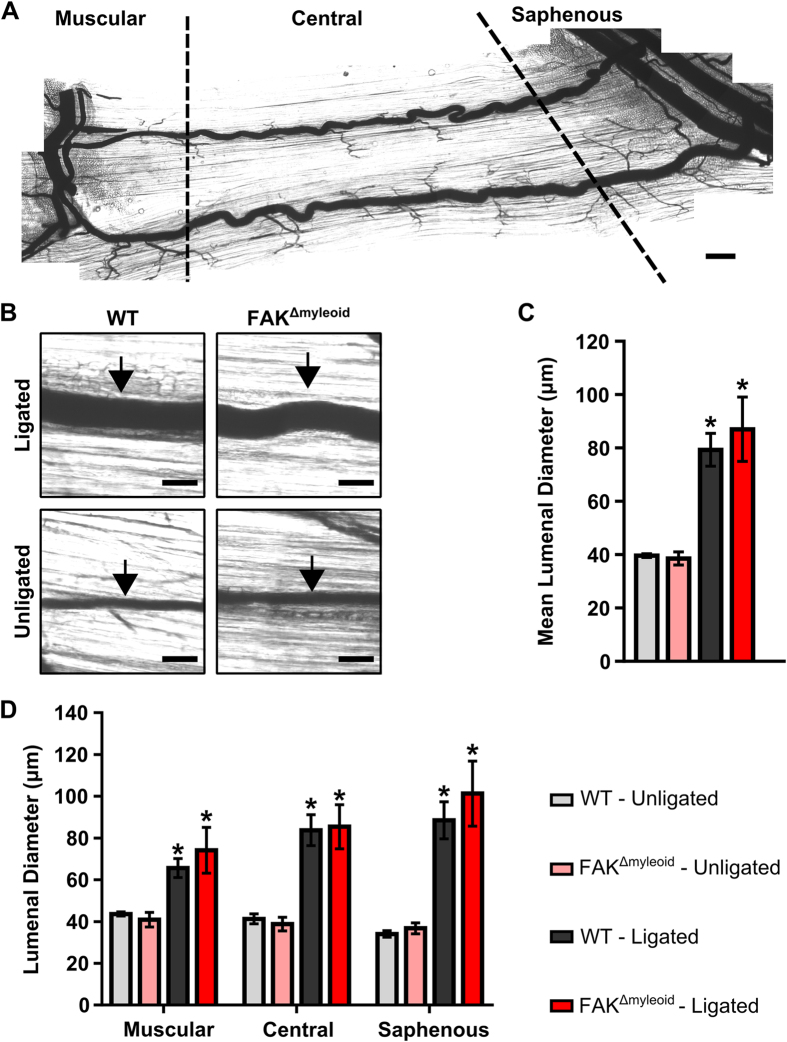
Arteriogenesis in the gracilis adductor muscle is independent of macrophage-specific FAK. (**A**) Representative image of gracilis muscle whole mount preparation 28 days post-FAL indicating muscular, central, and saphenous regions of analysis separated by dotted lines. Scale bar = 500 μm (**B**) Representative images from central region of gracilis muscle whole mount preparations from the ligated and unligated limbs of FAK^Δmyeloid^ and WT mice 28 days after FAL. Collateral arteries are identified by black arrows. Scale bar = 100 μm. (**C**) Bar graphs of mean lumenal collateral diameter averaged across all regions (muscular, central, and saphenous) and (**D**) in each region (muscular, central, and saphenous) between FAK^Δmyeloid^ and WT mice. There were no statistical differences between FAK^Δmyeloid^ and WT mice (n = 6). *p < 0.05 between ligated and unligated limbs within the given region.

We then sought to assess the role of myeloid-specific deletion of FAK on arteriogenesis of the collateral artery pathways within the gracilis adductor muscle. A vascular casting agent was perfused throughout the body for the identification and measurement of lumenal diameter along the length of the collateral artery (Fig. 2A). As expected, femoral arterial ligation induced significant outward remodeling of collateral arteries in ligated limbs (i.e. arteriogenesis) from baseline in control mice (79.3 ± 6.2 vs. 39.6 ± 0.7 μm; ligated vs. unligated averaged across all collateral regions, p < 0.05) (Fig. 2B,C). Myeloid-specific FAK knockout mice also demonstrated significant arteriogenesis in ligated limbs compared to unligated limbs (87.0 ± 12.0 vs. 38.6 ± 2.4 μm; ligated vs. unligated averaged across all collateral regions, p < 0.05) (Fig. 2B,C). As we have previously shown that the extent of arteriogenesis can vary depending on the collateral region^13^, we further examined collateral artery growth in each of the muscular, central, and saphenous regions. However, both FAK^Δmyeloid^ and WT control mice experienced a similar degree of outward remodeling in the ligated limb 28 days after FAL (65.6 ± 4.5 vs. 74.2 ± 11.0 μm, 83.8 ± 7.4 vs. 85.4 ± 10.6 μm, and 88.5 ± 8.9 vs.101.3 ± 15.6 μm in WT control vs. FAK^Δmyeloid^ mice in muscular, central, and saphenous regions respectively) (Fig. 2D). There were no significant differences between FAK^Δmyeloid^ or control mice in any region of the gracilis collateral arteries (n = 6 and n = 7, respectively).

### Conditional macrophage-specific FAK deletion affects neither angiogenesis nor skeletal muscle fiber morphometry

**Figure 3:**
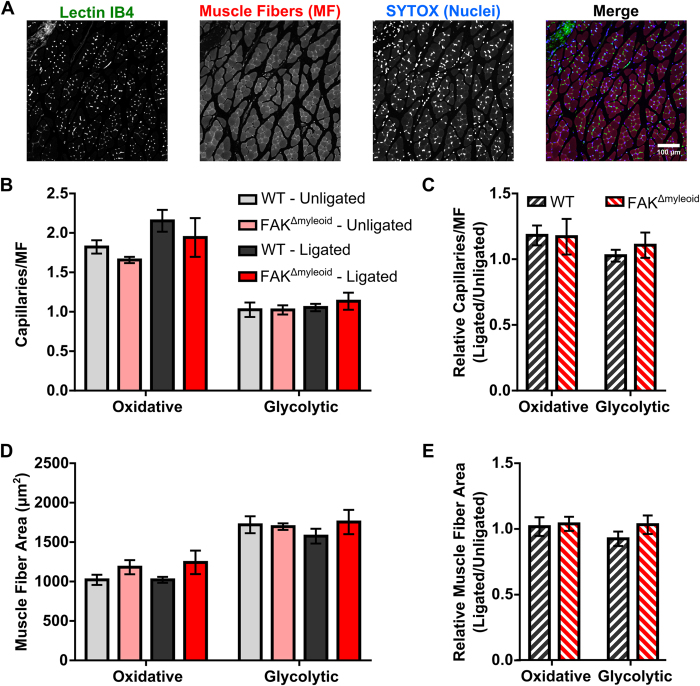
Conditional macrophage-specific FAK deletion affects neither angiogenesis nor skeletal muscle fiber morphometry within gastrocnemius after FAL. (**A**) Representative images from the oxidative region of the gastrocnemius muscle of the unligated limb of FAK^Δmyeloid^ mice immunolabeled with lectin-B4 (capillaries), muscle fibers, and nuclei. Scale bar = 100 μm. (**B**) Bar graph of absolute capillary to muscle fiber ratio, (**C**) relative capillary to muscle fiber ratio, (**D**) absolute muscle fiber size, and (**E**) relative muscle fiber size in the oxidative (plantaris and deep gastrocnemius) and glycolytic (superficial gastrocnemius) regions of FAK^Δmyeloid^ and WT mice. There were no statistical differences between FAK^Δmyeloid^ and WT mice (n = 6 and n = 5, respectively).

To investigate the role of myeloid-specific FAK deletion on neovascularization and muscle fiber remodeling, capillary and muscle fiber structures were analyzed in cross sections of the gastrocnemius 28 days after FAL (Fig. 3A). Within the calf muscle, there is a distinct variation in muscle fiber type^32^,^33^. For this reason, our analysis was divided into two regions: the glycolytic region (superficial, white gastrocnemius, predominantly IIB and IIDB fibers) and the oxidative region (deep, plantaris and red gastrocnemius muscle, mixed IID, IIA, and I fibers)^33^,^34^. As expected, glycolytic regions showed lower capillary-to-muscle fiber ratio (1.02 ± 0.09, WT; 1.02 ± 0.06, FAK^Δmyeloid^) and larger average fiber size (1720 ± 107 μm^2^, WT; 1696 ± 42 μm^2^, FAK^Δmyeloid^) than oxidative regions at baseline (unligated limb) (1.82 ± 0.08 capillary:fiber and 1021 ± 64 μm^2^, WT; 1.66 ± 0.04 capillary:fiber and 1182 ± 90 μm^2^, FAK^Δmyeloid^). In both the oxidative and glycolytic regions, there were no significant differences in capillary-to-muscle fiber ratio or muscle fiber area between unligated and ligated limbs in either WT control or FAK^Δmyeloid^ mice. Moreover, FAK^Δmyeloid^ mice and WT control mice displayed no significant differences in in capillary-to-muscle fiber ratio or muscle fiber area (Fig. 3B–E, n = 6 and n = 5, respectively).

### FAK is up-regulated upon differentiation from monocyte to macrophage

We have shown that conditional myeloid-specific deletion of FAK has no significant effects on relative perfusion recovery, arteriogenesis, or angiogenesis following femoral arterial ligation. We therefore conclude that vascular growth adaptations to chronic arterial occlusion occur independent of FAK expressed by myeloid cells. To this end, there are several possible explanations for why myeloid-specific deletion of FAK had no effect on vascular growth and perfusion recovery. One such explanation could be due to differential expression of FAK in distinct myeloid-cell populations.

Blood monocytes are crucial to arteriogenesis^31^, however there are two known populations of blood monocytes with distinct migratory roles during inflammation in mice: Ly6C^hi^ inflammatory monocytes and Ly6C^lo^ patrolling, resident monocytes^35^. Inflammatory monocytes (Ly6C^hi^/CX3CR1^lo^/CCR2^hi^) are recruited to sites of active inflammation through endothelial surface expression of the CCL2 ligand^35^. This subpopulation and the CCR2 ligand are well known to be up-regulated following femoral arterial ligation and to be critical for coordinating arteriogenesis^3^,^9^,^36^,^37^,^38^,^39^,^40^. In contrast, resident (Ly6C^lo^/CCR2^lo^/CX3CR1^hi^) monocytes exhibit a patrolling behavior with a large intravascular population that crawls along the venous endothelium, rapidly extravasating to sites of inflammation. The role of these patrolling monocytes in vascular remodeling is less clear. In one study, resident monocytes were not recruited to the ischemic hindlimb following femoral arterial ligation^38^. Furthermore, adoptive transfer of Ly6C^lo^ monocytes did not alter perfusion recovery or angiogenesis in contrast to adoptive transfer of Ly6C^hi^ monocytes^38^,^41^. However, other studies have implied a role of the Ly6C^lo^ monocyte population in both angiogenesis and arteriogenesis^42^,^43^,^44^,^45^.

**Figure 4:**
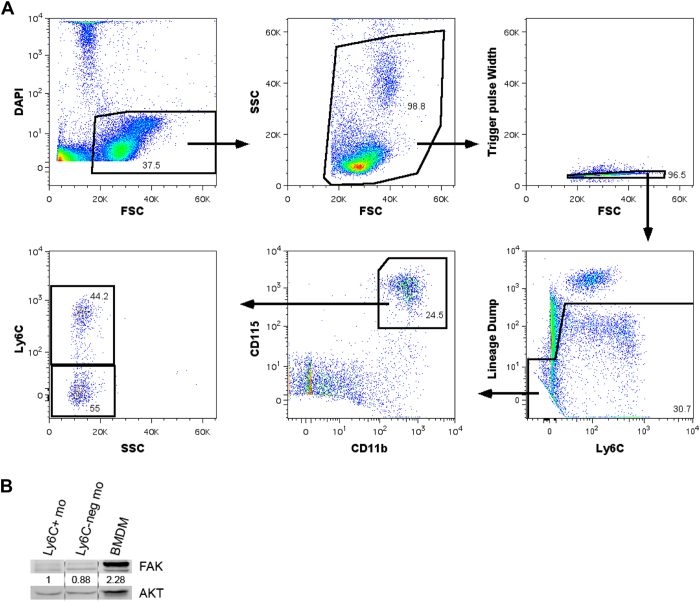
FAK is expressed at low levels in blood monocytes but is elevated in differentiated macrophages. (**A**) Schematic of cell sorting gating procedure. Monocytes were identified as lineage negative (lineage defined as CD49b, CD3e, CD19, and Ly6G positive for NK cells, T cells, B cells, and neutrophils respectively), CD11b^+^, and CD115^+^. (**B**) Western blot of FAK expression in Ly6C^+^ monocytes (Ly6C^+^ mo), Ly6C-negative monocytes (Ly6C^neg^ mo), and bone marrow derived macrophages (BMDM). Ly6C^+^ monocytes and Ly6C-negative monocytes were isolated from wild-type mice via the cell sorting protocol described in (**A**). BMDMs were isolated from whole bone marrow and cultured *in-vitro* for 7 days in αMEM base media supplemented with 10% FBS, penicillin/streptomycin, and 10% CMG 14–12 conditioned media.

We therefore sought to determine FAK expression in both Ly6C^hi^ inflammatory monocytes and Ly6C^lo^ patrolling, resident monocytes. To achieve this, monocytes were identified as lineage negative, CD11b^+^, and CD115^+^. This subset was then sorted further by Ly6C expression (Fig. 4A). We found that FAK is expressed, albeit at low levels, similarly both in blood inflammatory (Ly6C^hi^) monocytes and patrolling (Ly6C^lo^) monocytes (Fig. 4B). Additionally, we found that cultured bone marrow derived macrophages demonstrated approximately 2-fold greater FAK expression than Ly6C^hi^ and Ly6C^lo^ monocytes (Fig. 4B) suggesting that FAK expression typically increases as monocytes differentiate to macrophages. As FAK is expressed at such low levels in these monocytes, we would expect for FAK deletion to not significantly impact monocyte motility of either of these subpopulations. This is in contrast with the previous study demonstrating that FAK deletion impaired thioglycate-stimulated migration of peritoneal CD11b^+^ cells. However, this may indicate that the necessity of FAK may depend on the inflammatory stimulus and/or the basal expression level of FAK as inflammatory peritoneal CD11b^+^ cells expressed FAK at a relatively high levels compared to the low levels we observed in Ly6C^+^ peripheral blood monocytes^28^. Overall, our results indicate that FAK expression is not required for monocyte migration into the perivascular space or for the pro-arteriogenic functions of transdifferentiated macrophages, as constitutive deletion of FAK from these cells does not impair vascular remodeling.

Alternatively, myeloid-specific deletion of FAK may have no significant effects on vascular remodeling following femoral arterial ligation due to the presence of compensatory and/or FAK-independent mechanisms. To this end, while FAK deletion has been shown to significantly impair macrophage motility *in-vitro*, it does not abolish it^28^. Pyk2, the second member of the FAK family of phosphotyrosine kinases, is also expressed in the monocyte/macrophage lineage and is a known regulator of macrophage motility^28^,^46^. In acute, *in-vitro* experiments, endogenous Pyk2 is equivalently expressed in macrophages isolated from WT and FAK^Δmyeloid^ mice, but is unable to fully compensate for the motility defects due to the absence of FAK in these macrophages^28^. However, given the longer duration of our experiments, we cannot rule out the possibility that Pyk2 signaling is able to compensate for the loss of FAK. Additionally, signaling pathways independent of FAK/Pyk2 could regulate monocyte migration and subsequent vascular remodeling. Src, α4β1 integrins, and paxillin have been previously shown to regulate CSF-1 dependent motility in macrophages, independent of FAK^49^. Increased activation of these signaling pathways in monocytes could compensate for the loss of myeloid-specific FAK such that sufficient monocyte migration into the pericollateral space can occur enabling for normal vascular remodeling following femoral arterial ligation. Future work could examine the differential migratory mechanisms between Ly6C^lo^ and Ly6C^hi^ monocytes and whether myeloid-specific deletion of these alternative pathways impairs perfusion recovery, arteriogenesis, and/or angiogenesis.

## Methods

### Ethics Statement

All animal protocols were approved by the Institutional Animal Care and Use Committee at the University of Virginia (ACUC ID 3937 and 3158) and conformed to all regulations for animal use outlined in the American Heart Association Guidelines for the Use of Animals in Research. All surgery was performed under ketamine/xylazine/ atropine anesthesia. Animals were given analgesic post-operatively and all efforts were made to minimize suffering.

### Mice

Myeloid specific FAK knockout (FAK^Δmyeloid^) and littermate control mice (WT) were produced and genotyped as previously described^28^. Male mice 12–14 weeks of age with similar body weights were used for experiments. All animals were housed in the animal facilities at the University of Virginia.

### Femoral Arterial Ligation

To produce uniform hemodynamic changes in the collateral arteries in the superficial gracilis adductor muscle, we used a previously detailed femoral artery ligation scheme^13^,^50^. This particular ligation pattern has been shown to produce consistent arteriogenesis in the gracilis collateral arteries^7^,^8^,^13^,^51^,^52^,^53^,^54^ with minimal heterogeneity in the baseline collateral structure and with the predicted changes in flow direction from baseline. Male mice, 12–14 weeks of age, were anesthetized (i.p 120 mg/kg ketamine, 12 mg/kg xylazine, and 0.08 mg/kg atropine), depilated, and prepped for aseptic surgery. Mice were kept on a surgical heating pad immediately after anesthesia, throughout preparation and surgery, until recovery. On the left leg, an incision was made directly above and along the femoral artery, which was gently dissected from the femoral vein and nerve between the bifurcation of the superior epigastric artery and popliteal artery. Two 6.0 silk sutures were placed immediately distal to the epigastric artery, which served as the origin of the muscular branch artery in all mice, and the artery segment between the two ligatures was then severed with micro dissecting scissors. The surgical site was then closed with 5.0 prolene sutures. A sham surgery, wherein the femoral artery was exposed but not ligated, was performed on the right hindlimb (i.e. on the other leg). Animals received one injection of buprenorphine for analgesia at the time of surgery and a second dose 8–12 hours later.

### Laser Doppler Perfusion Imaging

For monitoring blood flow recovery and post-surgical ischemia, mice were anesthetized via 1.5% isofluorane delivered under constant oxygen. Mice were placed in a prone position and the soles of the feet were scanned (PeriCam PSI, PeriMed, Stockholm, Sweden). Mean foot perfusion was used to calculate relative perfusion ratio (ligated/unligated).

### Tissue Harvesting of Gracilis and Gastrocnemius Muscles

For analysis of lumenal diameters in the gracilis collateral arteries, vascular casting was performed using an opaque polymer that allows for accurate lumenal diameter measurements^53^. 28 days after femoral artery ligation, mice were anesthetized (i.p 120 mg/kg ketamine, 12 mg/kg xylazine, and 0.08 mg/kg atropine), then euthanized via an overdose of pentobarbital. The abdominal aorta was cannulated and the lower body was then perfused with 2% heparinized saline with 2 mmol/L adenosine (16404, Fisher Scientific, Pittsburg, PA) and 0.1 mmol/L papaverine (P3510, Sigma Aldrich, St Louis, MO) to clear and vasodilate the downstream vasculature at a constant rate of 1 mL/min (PHD2000, Harvard Apparatus) and then fixed with 4% paraformaldehyde solution (19943, Affymetrix, Cleveland, OH). The lower body was then perfused with 0.8 mL of the casting agent Microfil® (FlowTech, Inc, Carver, Massachusetts) at a constant pressure of 100 mmHg. Viscosity of Microfil® was adjusted to minimize transport across capillaries. After curing for 2 hours at room temperature, both the gracilis and gastrocnemius muscles were dissected free and then cleared in 50% glycerol in phosphate buffered saline (PBS) at 4 °C overnight.

### Collateral Network Analysis

Cleared gracilis muscles were mounted between two coverslips using 500 μm thick spacers (645501, Grace Bio-Labs Inc) to keep constant thickness. Muscles were imaged using transmitted light at 4x magnification on a Nikon TE200 inverted microscope with a CCD camera (Quantifier, Optronics Inc). Individual fields of view were montaged together (Photoshop CS2, Adobe Systems Inc). For analysis of lumenal diameter from intact gracilis collateral whole mounts (i.e. vascular casting), collateral regions were defined according to the following method. A cropped portion (512 pixel × 512 pixel) of the montaged image (previously randomized and de-identified) was taken of the collateral artery at muscular branch entrance region, the central region, and the saphenous region. This was done for each primary collateral running through the anterior and posterior heads of the gracilis muscle, yielding 6 total image regions per tissue whole mount. After each cropped image region was taken, all images were randomized and de-identified. The mean regional diameter was then taken from five separate diameter measurements along the length of cropped portion of the collateral artery.

### Immunofluorescence and Capillary Density Analysis

Sections (5 μm) of formalin-fixed, paraffin embedded gastrocnemius muscles were deparaffinized, rehydrated, then blocked in Carbofree blocking solution (1:10, Vector Labs, Burlingame, CA). Slides were then labeled with fluorophore conjugated primary antibody (isolectin-IB4-AlexFluor-647, 1:200, Life Technologies, Grand Island, NY) for 1 hour at room temperature. Nuclei were counterstained with Sytox green (500 uM, Life Technologies). Slides were washed and sealed with Prolong Gold (Life Technologies) to minimize photobleaching. Cross sections immunolabeled with isolectin-B4 were used to determine capillary density metrics. Analysis of the gastrocnemius muscle was separated into 2 distinct regions, termed here as the glycolytic (superficial, white gastrocnemius muscle) and oxidative regions (plantaris and deep, red gastrocnemius muscle), as they are composed of significantly different capillary and muscle fiber composition. Two fields of view from each region in each section were imaged at 20× magnification on a Nikon TE2000 C1 laser scanning confocal microscope. The number of capillaries (Isolectin-B4^+^ vessels, <7 um in diameter), muscle fibers (identified from autofluorescence), and muscle area were determined in each field of view using Fiji image analysis software. Each field of view yielded >100 muscle fibers.

### Harvest and Preparation of Cell Suspensions from Mouse Tissues

Mice were euthanized and tissues harvested in the following order. First, blood (500-700 μl/mouse) was drawn through cardiac puncture and placed in 1 ml of 5 mM EDTA/Hank’s balanced saline solution (HBSS) without magnesium or calcium. HBSS (10 ml) was then added to the suspension, the cells were centrifuged, and the cell pellet was resuspended in MACS buffer (0.5% BSA, 250 mM EDTA in PBS). Next, the spleen was placed in 10% FBS/DMEM, homogenized between glass microscope slides, washed in MACS buffer, and filtered through a 30 μm filter. Finally, bone marrow (BM) was flushed from the femurs and tibias with MACS buffer, washed in MACS buffer, and filtered through a 30 μm filter. To remove erythrocytes, the tissues were incubated in ammonium/chloride/potassium (ACK) lysis buffer (155 mM NH_4_Cl, 10 mM KHCO_3_, 0.1 mM Na_2_ EDTA 2H_2_O in H_2_O) for 5 min. at room temperature, quenched with complete media, and washed in MACS buffer.

### *In Vitro* Generation of Bone Marrow Derived Macrophages

Whole bone marrow was collected as described above, enumerated by hemacytometer, and added to the following media preparation at 4–6 × 10^6^ cells per 10 cm plate. Base media (αMEM with 10% FBS and penicillin/streptomycin) was supplemented with 10% CMG 14-12 conditioned media (source of CSF-1). Media was replaced every 3–4 days, and cells were cultured for seven days before harvesting for western blotting.

### Cell Sorting

Blood cell suspensions were prepared as described above and incubated with the FC blocking antibody α-CD16/32 (eBioscience, San Diego, CA; [5 μg/ml]) for 10 min. The cells were subsequently incubated with the following antibody panel for 25 mins. on ice: α-F4/80-AF488 (AbD serotec, Oxford, UK; [0.5 μg/ml]), α-CD115-PE (Biolegend, San Diego, CA; [1 μg/ml]), α-CD11b-PE/Cy7 (Biolegend; [0.5 μg/ml]), α-Ly6C-PerCP/Cy5.5 (BioLegend; [0.5 μg/ml]), α-Ly6G-APC (BioLegend; [1 μg/ml]), α-CD3e-APC (eBioscience; [1 μg/ml]), α-CD19-APC (eBioscience; [1 μg/ml]), α-CD49b-APC (eBioscience; [1 μg/ml]), and DAPI (Sigma; [0.1ug/ml]). Samples were stained concurrently with fluorescence minus one (FMO) antibody panels. Following a series of washes, the cells were resuspended in MACS buffer, and sorted on the BD Influx Cell Sorter (Becton, Dickinson and Company, Franklin Lakes, NJ). FlowJo software (Tree Star Inc., v.9.2, Ashland, OR) was used for data analysis.

### Western Blotting

Cell suspensions were washed in PBS, pelleted, incubated in RIPA lysis buffer, and immunoblotting was performed as described previously^28^. The following antibodies were utilized: α-FAK C-20 (Santa Cruz, Dallas, TX; [0.2 μg/ml]), α-ERK p44/42 (Cell Signaling, Danvers, MA; [1:2000]), and α-AKT (Cell Signaling; [1:1000]).

### Statistics

All results are reported as mean ± standard error. All data were first tested for normality. Statistical significance was assessed by two-way ANOVA with repeated measures for relative perfusion data and weight change data (Fig. 1B,C) and two-way ANOVA for lumenal diameter (Fig. 2C,D), capillary/muscle fiber (Fig. 3B), and muscle fiber area (Fig. 3D) data followed by paired comparisons using the Holm-Sidak method for multiple comparisons. Student *t* test was used for comparing relative capillary/muscle fiber ratio (Fig. 3C) and relative muscle fiber area (Fig. 3E) of WT vs. FAK in the oxidative and glycolytic regions (SigmaStat 3.5, Systat Inc). Significance was assessed at p < 0.05.

## Additional Information

**How to cite this article**: Heuslein, J. L. *et al*. Vascular growth responses to chronic arterial occlusion are unaffected by myeloid specific focal adhesion kinase (FAK) deletion. *Sci. Rep*. **6**, 27029; doi: 10.1038/srep27029 (2016).
